# Caregivers’ Attitude towards People with Mental Illness and Perceived Stigma: A Cross-Sectional Study in a Tertiary Hospital in Nepal

**DOI:** 10.1371/journal.pone.0158113

**Published:** 2016-06-23

**Authors:** Dipika Neupane, Sarmila Dhakal, Sabita Thapa, Parash Mani Bhandari, Shiva Raj Mishra

**Affiliations:** 1 Maharajgunj Medical Campus, Institute of Medicine, Kathmandu, Nepal; 2 School of Population Health, University of Western Australia, WA 6009, Perth, Australia; 3 Nepal Development Society, Bharatpur-10, Nepal; The George Institute for Global Health, INDIA

## Abstract

**Background:**

Mental illness is stigmatized in most of the communities and people with such illness are often subjected to defame. Stigma impairs an individual’s and their caregiver’s physical, social and emotional wellbeing, and health-seeking behavior. Sufficient literature on how often the caregivers of people with mental illness from low and middle-income countries are stigmatized and how they perceive people with mental illness is unavailable. In this study, we examined caregivers’ attitude towards people with mental illness and perceived stigma.

**Methods:**

We conducted face-to-face interviews with 170 caregivers in an outpatient clinic of a hospital in Nepal using a structured questionnaire. We calculated median and inter-quartile range of the attitude and perceived stigma scores. To assess the correlates, Kruskal Wallis H test and Mann Whitney U test were carried out.

**Results:**

Overall median score for the domains: attitude (score range: 18–90) and perceived stigma (score range: 12–60) were 42 and 28 respectively, inter-quartile range being 8 each. Attitude score differed significantly by the sex of caregiver (p<0.05), educational status of caregiver (p<0.001), sex of patient (p<0.05) and type of mental illness (p<0.05). Perceived stigma score varied significantly by caregiver’s sex (p<0.05), marital status (p<0.001), educational status (p<0.001), occupation (p<0.05), relation with the patient (p<0.005) and use of alternative treatment modalities (p<0.05).

**Conclusion:**

Sex of participant, educational status, sex of patient and type of mental illness were the correlates of attitude towards mental illness. Similarly, sex of participant, marital status, educational status, occupation, caregiver’s relation with patient and use of alternative treatment modalities were correlates of perceived stigma. Findings of this study suggest that interventions targeting these high-risk populations might be beneficial to help build a positive attitude and overcome the perceived social stigma.

## Introduction

People With Mental Illness (PWMI) are stigmatized in most of the societies, the intensity being more profound in the Asia [1]. Disgrace to these people is so high that even their caregivers face its spillover effects—referred to as “courtesy stigma” [2]. The stigma contributes to negative consequences for both patient and caregivers perpetuating self-stigmatization and low self-esteem [3, 4]. PWMI are discriminated and isolated [5, 6], while caregivers avoid social interactions and face social exclusion [7]. When stigma exists, the caregiver may conceal their relationship with patient, fail to acknowledge the illness and avoid treatment of the patient. Also, attitude towards mental illness shapes the way PWMI are treated in a society. Negative attitude hinders social integration of these people [8, 9], whereas positive attitude held by caregivers provides support to the patient in prevention, early treatment and rehabilitation [10].

Globally, both patients and their caregivers suffer stigma [11, 12] and are often deprived of their basic human rights [13]. Consistent with the global experience, PWMI and their caregivers in Asia are treated unequally [1, 14]. Due to the stigma, patients and their caregivers in Asia are hesitant to visit psychiatric facilities for treatment of mental illness [15]. On one hand, stigma tends to avoid or delay health seeking behavior increasing their vulnerability to violence, exploitation, malnutrition, drug abuse, and even suicide and death [16, 17]. On the other hand, stigma leads to numerous detrimental consequences from economic and social perspective, and has tremendous impact on family relations and employment affecting the quality of life of PWMI and their caregivers [5, 18, 19].

Only few studies on mental health services and stigma are available from Nepal [20–22]. In a qualitative formative study conducted in Nepal, attitude of community towards PWMI was observed to be highly negative and aggravated by denial and hatred [21]. Though the role of caregivers in successful treatment of patient is sufficiently proven to be of great significance, to our knowledge, study on caregivers’ attitude towards PWMI and perceived stigma is non-existent in Nepal.

In this paper, we report caregivers' attitude towards PWMI (diagnosed with a mental illness—organic disorders, schizophrenia, mood disorders, neurotic, stress-related and somatoform disorders, behavioral syndromes, mental retardation or other diseases of nervous system), and their perceived stigma regarding mental illness. We also assess the factors that contribute to difference in attitude and perceived stigma among caregivers.

## Methods

### Study setting

This is a cross-sectional study conducted in Kathmandu, the capital and most populous city of Nepal. We recruited caregivers of outpatients with recorded mental illness from Department of Psychiatry, Tribhuvan University Teaching Hospital (TUTH). TUTH is a site for seeking psychiatric health services at an affordable cost, and has a regular flow of patients from different parts of the country.

### Measures

For the assessment of perceived stigma, we developed a questionnaire based on items from Internalized Stigma of Mental Illness (ISMI) and an earlier study by Ostman et al [4, 23], while that for attitude was adapted from Community Attitude towards Mental Illness (CAMI) tool [24]. Both the ISMI and CAMI were not designed specifically to assess caregiver’s attitude in a hospital-based setting, so we selected only the items from these tools that were relevant to our study objectives (for details, see questionnaire items- S1 File). The items on stigma measured the intensity of caregivers being stereotyped and the items on attitude reflected caregivers’ individual attitude towards PWMI. Response to each of these items was recorded in a scale of 1–5. A bilingual translator translated the questionnaire into Nepali language. We pre-tested the questionnaire among all the caregivers visiting the outpatient department on 23^rd^ April 2014 (n = 10) of TUTH. These participants were not included in the main study. Based on the pretesting, questionnaire sequence was organized, language was modified and few questions with repetitive responses were removed.

We recorded information on caregivers' sex (Male or Female), religion (Hindu, Buddhist or Others), residence (Rural or Urban), marital status (Married or Unmarried), educational status (Illiterate, Primary level, Secondary level or Higher), type of family (Nuclear or Extended), occupation (Agriculture, Business, Service, Foreign employment or Others) and relation with patient (Parents, Spouse, Children, Siblings, In-laws or Others). Caregivers belonging to a Village Development Committee (a relatively rural area with population below 20,000 and lacking basic infrastructures) were grouped as rural residents and those from municipality (a relatively urban area with population above 20,000 and having basic infrastructures) were grouped as urban residents. Family that constituted only a couple and their children was grouped as a nuclear family while other types of families were categorized as an extended family.

Patients’ information included sex (Male or Female) and age (15–24 years, 25–34 years, 35–44 years, 45–54 years or ≥55 years). Type of mental illness of the patient was categorized based on ICD-10 classification (Organic disorders, Schizophrenia, Mood disorders, Neurotic, stress-related and somatoform disorders, Behavioural syndromes, Mental retardation or Other diseases of nervous system) [25]. Duration of illness was recorded as the duration between interview and the date of diagnosis. It was categorized into four groups (<1 year, 1–5 years, 5–10 years or ≥10 years) for statistical analysis. Information regarding the treatment site used in first visit was recorded; response to this had two alternatives (Traditional or Modern). Information on the use of treatment modalities other than the allopathic was also recorded (Yes or No).

### Procedure

The data were collected from 170 caregivers visiting Outpatient Department of Department of Psychiatry, TUTH between May and July 2014. Caregivers below 18 years of age were excluded from our study due to ethical reasons as it was not feasible to take parental consent in the Outpatient Department.

Caregivers of patients who visited during the study period were approached for face-to-face interviews. In the process of recruitment, we first checked the Outpatient Card of patient for confirmation of a mental illness. Each card in the reception was checked and the patient was then followed. Caregivers of the patient were identified and approached when they accompanied the PWMI for outpatient consultation. Among those approached, 29 caregivers (14.57%) denied to participate in the study. Finally, 170 caregivers were then interviewed in the waiting room placing them at a distance from the patient. Authors themselves (DN, SD and ST) conducted all the face-to-face interviews.

### Data analysis

Data were entered in EpiData 3.1 and was analyzed using SPSS version 17 (SPSS, Chicago, IL, USA). Negatively worded items were first reverse-coded so that for every item higher score reflected negative attitude or greater perceived stigma. Scores were then summed up to yield a composite attitude score and perceived stigma score. The practice of summing of the scores helps in better comprehension of the difference in the scores between subgroups and is common in studies assessing stigma or attitude [26, 27]. Possible score for attitude was 18–90, in which higher score meant a more negative attitude. Perceived stigma score could range from 12–60 where a higher score implied greater perceived stigma.

For descriptive statistics, frequency and percentage of each demographic characteristics were calculated. Median and inter-quartile range were also calculated for each item on attitude and perceived stigma scale. Shapiro Wilk test of the composite scores showed non-normal distribution so we used non-parametric tests for further analysis [28]. We then computed median score for each category, performed the Mann–Whitney U test or Kruskal Wallis H test to compare the scores between groups and Dunn-Bonferroni tests for post-hoc comparisons [29]. P-value less than 0.05 were considered statistically significant.

### Ethics

The study was approved by Institutional Review Board, Institute of Medicine, Tribhuvan University. We obtained prior permission from the hospital administration for the conduction of research. Participants were explained about study objectives and written consent was obtained. Participants received no incentive for participation into this study.

## Results

Out of 170 participants interviewed, 52.0% were male and 72.5% were ever-married. Further, 45.6% were from rural background and the major occupation of participants was service (39.8%) followed by agriculture (30.4%). The majority of caregivers were Hindu (87.1%) and 91.7% were literate. The majority of the patients (65.5%) were female and most of them were accompanied by parents (23.4%) followed by siblings (21.6%) and children (son/daughter) (21.1%). Three most common diagnoses of the patients were mood disorders (34.5%), neurotic, stress-related and somatoform disorders (26.9%) and schizophrenia (22.2%). The most common point of first contact for treatment was found to be modern/allopathic treatment (71.9%). Very few (15.2%) patients were found to be treated with ways of treatment other than allopathic treatment.

### Correlates of attitude towards PWMI and perceived stigma

Significant difference in median attitude score by sex of caregiver (p<0.05) and sex of patient (p<0.05) was observed. Median attitude score when compared with the educational status of caregiver, a significant difference in scores between different groups was observed (p<0.001). Post hoc test showed significant difference in attitude scores between caregivers with higher education and those with primary education (p<0.001), and caregivers with higher education and illiterates (p<0.005). Similarly, median attitude score was significantly different between different types of mental illness (p<0.05). Post hoc test revealed difference in attitude score between the caregivers of patient having mood disorders and those having schizophrenia (p<0.05).

We observed a significant difference in median perceived stigma score by the sex of caregiver (p<0.05) and marital status of caregiver (p<0.001). Median perceived stigma score varied significantly by the educational status of caregiver (p<0.001). Post hoc test showed difference in perceived stigma scores between caregivers with higher education and those with primary education (p<0.001), and caregivers with higher education and those who were illiterate (p<0.001). Occupation of caregiver showed a significant difference in median perceived stigma score (p<0.05). On post-hoc analysis, difference in perceived stigma between caregivers in ‘Service’ category and those in ‘Others’ category (p<0.05), and caregivers in ‘Agriculture’ category and those in ‘Others’ category (p<0.05) was revealed. Also, difference in median perceived stigma score between different relation of caregivers with patient was observed (p<0.001). Post hoc test showed difference between caregivers who were children and those who were parents (p<0.001), and caregivers who were siblings and those who were parents (p<0.005). Similarly, difference in median perceived stigma score between those who used alternative treatment modalities and those who didn’t use alternative treatment modalities was revealed (p<0.05). (Table 1)

**Table 1:** Socio-demographic characteristics in relation to attitude towards PWMI and perceived stigma.

Characteristics	Number (%)	Attitude towards PWMI		Perceived stigma	
		Median score	p-value	Median score	p-value
**Sex**					
Male	89 (52.0%)	41.00	**0.019**	27.00	**0.012**
Female	82 (48.0%)	43.00		29.00	
**Religion**					
Hindu	149 (87.1%)	42.00	0.587	28.00	0.481
Buddhist	17 (9.9%)	43.00		27.00	
Others	5 (2.9%)	43.00		29.00	
**Residence**					
Rural	78 (45.6%)	43.00	0.132	28.00	0.583
Urban	93 (54.4%)	41.00		28.00	
**Marital status**					
Married	124 (72.5%)	42.50	0.119	29.00	**<0.001**
Unmarried	47 (27.5%)	41.00		25.00	
**Educational status**					
Illiterate	15 (8.8%)	48.00	**<0.001**	34.00	**<0.001**
Primary level	29 (17.0%)	44.00		31.00	
Secondary level	37 (21.6%)	42.00		29.00	
Higher	90 (52.6%)	40.00		26.00	
**Family type**					
Nuclear	86 (50.3%)	42.00	0.814	28.50	0.438
Extended	85 (49.7%)	42.00		28.00	
**Occupation**					
Agriculture	52 (30.4%)	42.00	0.091	28.00	**0.036**
Business	28 (16.4%)	42.50		28.50	
Service	68 (39.8%)	41.00		27.50	
Foreign employment	11 (6.4%)	48.00		32.00	
Others	12 (7.0%)	44.00		32.00	
**Relation with patient**					
Parents	40 (23.4%)	43.50	0.210	32.00	**<0.001**
Spouse	33 (19.3%)	41.00		28.00	
Children	36 (21.1%)	42.00		25.00	
Siblings	37 (21.6%)	42.00		26.00	
In-laws	12 (7.0%)	46.00		30.00	
Others	13 (7.6%)	39.00		28.00	
**Sex of patient**					
Male	59 (34.5%)	43.00	**0.034**	29.00	0.190
Female	112 (65.5%)	41.00		28.00	
**Age of patient (years)**					
15–24	55 (32.2%)	42.00	0.974	28.00	0.523
25–34	36 (21.1%)	41.00		29.50	
35–44	26 (15.2%)	42.50		28.00	
45–54	32 (18.7%)	42.00		27.00	
≥55	22 (12.9%)	42.50		27.00	
**Type of illness**					
Organic disorders	16 (9.4%)	40.00	**0.010**	29.50	0.281
Schizophrenia	38 (22.2%)	44.00		29.00	
Mood disorders	59 (34.5%)	40.00		26.00	
Neurotic, stress-related and somatoform disorders	46 (26.9%)	43.00		28.50	
Behavioral syndromes	3 (1.8%)	36.00		24.00	
Mental retardation	3 (1.8%)	46.00		32.00	
Other diseases of nervous system	6 (3.5%)	40.00		28.50	
**Duration of illness (years)**					
<1	64 (37.4%)	44.00	0.252	28.00	0.662
1–5	59 (34.5%)	41.00		28.00	
5–10	24 (14.0%)	41.00		29.00	
≥10	24 (14.0%)	41.00		27.50	
**Treatment site consulted in first visit**					
Traditional	48 (28.1%)	42.00	0.405	29.00	0.725
Modern	123 (71.9%)	42.00		28.00	
**Use of treatment modality other than allopathy**					
Yes	26 (15.2%)	42.00	0.302	30.50	**0.015**
No	145 (84.8%)	42.00		28.00	

### Attitude towards PWMI

Overall median score and the inter-quartile range for attitude domain were 42 and 8 respectively. Upon analysis of individual items, the highest median attitude score (four) was found for three items: mentally ill people are violent, the mentally ill people have to be controlled like a young child, and person should be hospitalized soon after signs of a mental illness. Moreover, lowest median attitude score (one) was found for the statement that indicated that it is our responsibility to provide best care to PWMI. (Table 2)

**Table 2:** Median score of items measuring attitude towards PWMI.

SN	Item	Possible scores^#^	Median score	Inter-quartile range
1	Mental illness is like any other disease.	1–5	3	2
2	Virtually anyone can be mentally ill.	1–5	2	1
3	The mentally ill should not be denied of their individual right.	1–5	2	1
4	Mentally ill people are violent.*	1–5	4	2
5	Mentally ill are burden on the society.*	1–5	2	1
6	Mentally ill should be isolated from the rest of the community.*	1–5	2	1
7	The mentally ill should not be given any responsibility.*	1–5	2	2
8	Mental patient needs the same kind of control and discipline as a young child.*	1–5	4	2
9	The best way to handle the mentally ill is to keep them inside the locked doors.*	1–5	2	1
10	We have a responsibility to provide best possible care for the mentally ill.	1–5	1	1
11	The mentally ill doesn't deserve our sympathy.*	1–5	2	2
12	The mentally ill should not be treated as outcast of the society.	1–5	2	0
13	Having mental patient living within the residential neighbourhoods will be good therapy but the risks to residents are too great.*	1–5	2	1
14	We need to adopt far more tolerant attitude towards the mentally ill in our society.	1–5	2	1
15	As soon as a person shows signs of mental disturbance, he should be hospitalized.*	1–5	4	1
16	As far as possible mental health services should be provided through community based facilities.	1–5	2	1
17	There are sufficient existing services for the mentally ill.	1–5	2	1
18	Mental health facilities should be kept outside residential neighborhood.*	1–5	2	4

*items reverse scored

^#^1: totally agree, 2: agree, 3: neutral, 4: disagree, 5: totally disagree

### Perceived stigma towards mental illness

Overall median score and inter-quartile range for perceived stigma among caregivers were 28 and 8 respectively. Item that asked if the caregiver has felt supported by people in carrying the burden of having a relative with mental illness received greatest median stigma score (four)—meaning higher stigma as measured by that particular item. All items other than this received median score of two—suggesting lower perceived stigma. (Table 3)

**Table 3:** Median score of items measuring perceived stigma.

SN	Item	Possible scores^#^	Median	Inter-quartile range
1	I avoid telling people that I have a family member living with mental illness.*	1–5	2	4
2	Having a family member living with mental illness has spoiled my life.*	1–5	2	4
3	People having a family member with mental illness cannot live a good or rewarding life.*	1–5	2	2
4	There times when you wished that the person with mental illness had never been born or that you and the person had never met.*	1–5	2	1
5	You ever had the thought that it would be better if the mentally ill patient was dead off.*	1–5	2	1
6	The burden of the situation of being relatives was so heavy that you have ever thought of suicide.*	1–5	2	1
7	People often patronize me just because I have a family member with mental illness.*	1–5	2	0
8	Nobody would be interested in getting close to me just because I have family member with mental illness.*	1–5	2	0
9	I don’t socialize as much as I used to because my family member’s mental illness might make me look or behave wired.*	1–5	2	0
10	I feel supported by people in carrying the burden of having a relative with mental illness	1–5	4	2
11	People discriminate against me because I have a family member with mental illness.*	1–5	2	0
12	I find psychiatric services to be supportive in carrying the burden of being a relative of a person with mental illness.	1–5	2	0

*items reverse scored

^#^1: totally agree, 2: agree, 3: neutral, 4: disagree, 5: totally disagree

## Discussion

To our knowledge, this is the first study to assess attitude towards PWMI and perceived stigma among caregivers in Nepal. In this study, we found that attitude score differed by sex of caregiver, educational status, sex of patient and type of mental illness of PWMI. Experience of perceived stigma differed by sex, educational status, marital status, occupation, relation with the PWMI and use of treatment modalities other than allopathy.

We found that attitude score differed significantly between male and female caregivers. Literature on the relation of caregiver’s gender with attitude towards people with mental illness is inconsistent. Studies from India and Nigeria reported that female sex was not a predictor of poor attitude towards PWMI [30, 31], while findings of a study from England and Qatar claimed the other way round [32, 33]. This can be attributed to the gender roles as gender is a complex construct determined by culturally set conventions, practices and behaviours [34] and differs between nations.

Attitude score differed significantly between caregivers of different educational Status. Possibly, educated caregivers are better exposed to mass media and are more aware of myths about PWMI in comparison to their less-educated counterparts. In an earlier study, Angermeyer et al. reported better educated people try to maintain distance with PWMI, which in turn contributes to greater discrimination against PWMI [35].

Attitude towards PWMI differed between different types of mental illness. Similar to the findings of our study, Dalky reported that attitude towards the PWMI and their families differs by the type of chronic mental illness [36]. Since patients with some of the mental illnesses are often characterized by visible behavioural changes, such diseases might receive greater degree of negative attitude.

Perceived stigma score varied significantly between male and female caregivers. This difference can be attributed to difference in social roles- as females are often caregivers of ill family members, they might be at increased risk of perceiving stigma in comparison to males. We observed that perceived stigma score differed significantly by the caregiver’s relation with PWMI. This difference in stigma can be reasoned in light of the difference in perceived burden of care among the caregivers [37] that might influence the perceived stigma.

This study was conducted before the devastating earthquake on 25^th^ April, 2015 which killed nearly 8,600 and displaced at least 2.8 million people [38]. While trauma and distress during the earthquake is likely to increase cases of mental illness in the aftermath, our study only reports caregivers’ attitude towards PWMI and perceived stigma in the pre-earthquake scenario. Adequate attention to stigma and attitude attached with mental illness has become more important in the post-earthquake Nepal, as the burden of mental illnesses were already high before earthquake [39]. The findings of this study shall contribute to the literature on perceived stigma and attitude toward PWMI in other low- and middle- income countries.

### Limitations

Before interpreting the findings of this study, few limitations need to be given due consideration. Caregivers of patients seeking no treatment and admitted in the in-patient clinics were not the participants in our study; therefore our study sample might have excluded PWMI in earlier stage and PWMI with a relatively severe illness. This study population might not be the true representative of background-population where the study-findings can be referred to, as caregivers of patients visiting hospital might have better attitude and lower perceived stigma in comparison to those in community setting. Among the approached caregivers, 14.57% denied to participate which might have affected the findings. In addition, we did not carry out a multivariate analysis so the confounding effect of other variables has not been addressed. Given the large number of tests conducted on relatively small sample size, probability of type-1 error is high. Finally, as the assessment was based on instrument developed by taking items from ISMI and CAMI with contextual relevancy into consideration, and has not been validated previously, rooms to question the construct validity of the instrument does exist.

## Conclusion

This study concludes that caregiver’s attitude score differs by sex of caregiver, educational status, sex of patient and type of mental illness. Likewise, the perceived stigma score is different between sex of participant, marital status, educational status, occupation, caregiver’s relation with patient and use of alternative treatment modalities. Considering these correlates, interventions tailored to the needs of these high risk populations might be helpful in building a positive attitude towards PWMI among caregivers and overcoming the perceived stigma.

## Supporting Information

S1 File Questionnaire. (RTF)
