# Supplementary material for: Caregivers’ Attitude towards People with Mental Illness and Perceived Stigma: A Cross-Sectional Study in a Tertiary Hospital in Nepal
Source: PLoS One. 2016 Jun 23;11(6):e0158113. doi: 10.1371/journal.pone.0158113 (PMC4918972; doi:10.1371/journal.pone.0158113)
Supplement: S1 File — (RTF) [file pone.0158113.s001.rtf]

1. Sex (of respondent):	
[   ] Male
	[   ] Female
2. Religion (of respondent):	
[   ] Hindu
	[   ] Buddhist
	[   ] Others
3. Residence (of respondent):			
[   ] Rural
	[   ] Urban
4. Marital status (of respondent):		
[   ] Married
	[   ] Unmarried
5. Educational status (of respondent):		
[   ] Illiterate
	[   ] Primary level
	[   ] Secondary level
	[   ] Higher
6. Family type (of respondent): 		
[   ] Nuclear
	[   ] Extended
7. Occupation (of respondent):			
[   ] Agriculture
	[   ] Business
	[   ] Service
	[   ] Foreign Employment
	[   ] Others
8. Relation with patient (of respondent):	
[   ] Parents
	[   ] Spouse
	[   ] Children
	[   ] Siblings
	[   ] In-laws
	[   ] Others
9. Sex (of patient):		
[   ] Male
	[   ] Female
10. Age (of patient):		
…………years
11. Type of illness:		
[   ] Organic disorder
	[   ] Schizophrenia
	[   ] Mood disorders
	[   ] Neurotic, stress-related and somatoform disorders
	[   ] Behavioral syndromes
	[   ] Mental retardation
	[   ] Other diseases of nervous system
12. Duration of illness:	
………….years
13. Treatment site consulted in first visit:	
[   ] Traditional
	[   ] Modern
14. Using other treatment modalities:	
[   ] Yes
	[   ] No
15. Satisfied with hospital services:
[   ] Yes
	[   ] No
Domain-I: Attitude
IA1: Mental illness is like any other disease.
[   ] Totally agree
[   ] Agree
[   ] Neutral
[   ] Disagree
[   ] Totally disagree			
IA2: Virtually anyone can be mentally ill.
[   ] Totally agree
[   ] Agree
[   ] Neutral
[   ] Disagree
[   ] Totally disagree
IA3: The mentally ill should not be denied of their individual right.
[   ] Totally agree
[   ] Agree
[   ] Neutral
[   ] Disagree
[   ] Totally disagree	
IA4: Mentally ill people are violent.
[   ] Totally agree
[   ] Agree
[   ] Neutral
[   ] Disagree
[   ] Totally disagree	
IA5: Mentally ill are burden on the society.
[   ] Totally agree
[   ] Agree
[   ] Neutral
[   ] Disagree
[   ] Totally disagree	
IA6: Mentally ill should be isolated from the rest of the community.
[   ] Totally agree
[   ] Agree
[   ] Neutral
[   ] Disagree
[   ] Totally disagree	
IA7: The mentally ill should not be given any responsibility.
[   ] Totally agree
[   ] Agree
[   ] Neutral
[   ] Disagree
[   ] Totally disagree	
IA8: Mental patient needs the same kind of control and discipline as a young child.
[   ] Totally agree
[   ] Agree
[   ] Neutral
[   ] Disagree
[   ] Totally disagree	
IA9: The best way to handle the mentally ill is to keep them inside the locked doors.
[   ] Totally agree
[   ] Agree
[   ] Neutral
[   ] Disagree
[   ] Totally disagree	
IA10: We have a responsibility to provide best possible care for the mentally ill.
[   ] Totally agree
[   ] Agree
[   ] Neutral
[   ] Disagree
[   ] Totally disagree	
IA11: The mentally ill doesn't deserve our sympathy.
[   ] Totally agree
[   ] Agree
[   ] Neutral
[   ] Disagree
[   ] Totally disagree	
IA12: The mentally ill should not be treated as outcast of the society.
[   ] Totally agree
[   ] Agree
[   ] Neutral
[   ] Disagree
[   ] Totally disagree	
IA13: Having mental patient living within the residential neighbourhoods will be good therapy but the risks to residents are too great.
[   ] Totally agree
[   ] Agree
[   ] Neutral
[   ] Disagree
[   ] Totally disagree	
IA14: We need to adopt far more tolerant attitude towards the mentally ill in our society.
[   ] Totally agree
[   ] Agree
[   ] Neutral
[   ] Disagree
[   ] Totally disagree	
IA15: As soon as a person shows signs of mental disturbance, he should be hospitalized.
[   ] Totally agree
[   ] Agree
[   ] Neutral
[   ] Disagree
[   ] Totally disagree	
IA16: As far as possible mental health services should be provided through community based facilities.
[   ] Totally agree
[   ] Agree
[   ] Neutral
[   ] Disagree
[   ] Totally disagree	
IA17: There are sufficient existing services for the mentally ill.
[   ] Totally agree
[   ] Agree
[   ] Neutral
[   ] Disagree
[   ] Totally disagree	
IA18: Mental health facilities should be kept outside residential neighborhood.
[   ] Totally agree
[   ] Agree
[   ] Neutral
[   ] Disagree
[   ] Totally disagree	
Domain II: Stigma
IS1: I avoid telling people that I have a family member living with mental illness.
[   ] Totally agree
[   ] Agree
[   ] Neutral
[   ] Disagree
[   ] Totally disagree	
IS2: Having a family member living with mental illness has spoiled my life.
[   ] Totally agree
[   ] Agree
[   ] Neutral
[   ] Disagree
[   ] Totally disagree	
IS3: People having a family member with mental illness cannot live a good or rewarding life.
[   ] Totally agree
[   ] Agree
[   ] Neutral
[   ] Disagree
[   ] Totally disagree	
IS4: There times when you wished that the person with mental illness had never been born or that you and the person had never met.
[   ] Totally agree
[   ] Agree
[   ] Neutral
[   ] Disagree
[   ] Totally disagree	
IS5: You ever had the thought that it would be better if the mentally ill patient was dead off.
[   ] Totally agree
[   ] Agree
[   ] Neutral
[   ] Disagree
[   ] Totally disagree	
IS6: The burden of the situation of being relatives was so heavy that you have ever thought of suicide.
[   ] Totally agree
[   ] Agree
[   ] Neutral
[   ] Disagree
[   ] Totally disagree	
IS7: People often patronize me just because I have a family member with mental illness.
[   ] Totally agree
[   ] Agree
[   ] Neutral
[   ] Disagree
[   ] Totally disagree	
IS8: Nobody would be interested in getting close to me just because I have family member with mental illness.
[   ] Totally agree
[   ] Agree
[   ] Neutral
[   ] Disagree
[   ] Totally disagree	
IS9: I don't socialize as much as I used to because my family member's mental illness might make me look or behave wired.
[   ] Totally agree
[   ] Agree
[   ] Neutral
[   ] Disagree
[   ] Totally disagree	
IS10: I feel supported by people in carrying the burden of having a relative with mental illness.
[   ] Totally agree
[   ] Agree
[   ] Neutral
[   ] Disagree
[   ] Totally disagree	
IS11: People discriminate against me because I have a family member with mental illness.
[   ] Totally agree
[   ] Agree
[   ] Neutral
[   ] Disagree
[   ] Totally disagree	
IS12: I find psychiatric services to be supportive in carrying the burden of being a relative of a person with mental illness.
[   ] Totally agree
[   ] Agree
[   ] Neutral
[   ] Disagree
[   ] Totally disagree
